# Quantitative Assessment of Concrete Surface Topography: Effects of Mechanical Treatment and Measurement Resolution

**DOI:** 10.3390/ma18235320

**Published:** 2025-11-25

**Authors:** Slawomir Czarnecki

**Affiliations:** Faculty of Civil Engineering, Wroclaw University of Science and Technology, 50-370 Wroclaw, Poland; slawomir.czarnecki@pwr.edu.pl

**Keywords:** concrete surface, surface morphology, metrology, scale effect, mechanical treatment, 3D laser scanning, roughness

## Abstract

**Highlights:**

**What are the main findings?**

- Surface preparation strongly alters concrete surface morphology.
- Aggressive treatments increase roughness; moderate treatments smooth surfaces.
- The measurement scale affects parameter values; coarser scales reduce detail.

**What are the implications of the main findings?**

- Some parameters are robust to scale, suitable for comparative studies.
- Accurate surface characterization requires considering both treatment and scale.
- The findings guide a reproducible evaluation of concrete surfaces in engineering research.

**Abstract:**

Surface morphology strongly influences the performance and durability of concrete structures, yet the combined effects of mechanical preparation and measurement scale remain insufficiently quantified. This study analyzes three surface conditions, patched, ground, and shot-blasted, using 3D laser scanning measurements acquired at five spatial resolutions. Mechanical surface preparation was found to be the dominant factor shaping morphology: grinding reduced amplitude- and volume-related parameters by approximately 40–70%, while shot blasting increased them by 50–90%, producing highly textured surfaces with an expanded developed area. Measurement resolution additionally affected parameter magnitudes, with coarser sampling intervals reducing scale-sensitive descriptors such as *Sdr* and *Sdq* by more than 80–90%. In contrast, parameters including *Ssk*, *Sku*, *Smr1*, and *Smr2* varied by less than 5% across scales, demonstrating strong robustness. Patched surfaces exhibited the largest variability (coefficients of variation often exceeding 20–30%) due to manual finishing, whereas mechanically treated surfaces showed more uniform profiles. These quantitative results highlight the coupled influence of preparation method and measurement scale and provide practical guidance for reproducible surface characterization in engineering and material research.

## 1. Introduction

Concrete surface morphology plays a critical role in determining the functional performance of structural and repair applications [1,2]. Surface characteristics influence adhesion, durability, friction, and the overall interaction with coatings, overlays, and other applied materials [3,4]. The accurate and reliable characterization of surface morphology is therefore essential for both quality control in construction and the optimization of performance in practical applications [5]. Mechanical surface preparation is commonly employed to modify concrete surfaces and tailor their topography to specific functional requirements [6]. A variety of methods exist, ranging from moderate treatments, such as light grinding and controlled sand blasting, to aggressive treatments, including high-energy shot blasting and intensive milling [7]. Each treatment produces surfaces with distinct textural features, and the intensity of the process often determines the degree of roughness, micro-geometry alteration, and overall surface uniformity [8]. Considering the diversity of available techniques, it is important to systematically evaluate how different treatments influence the measured morphological characteristics and how they may affect downstream performance [9]. Surface preparation plays a fundamental role in ensuring the mechanical and durability performance of concrete–concrete interfaces in both structural strengthening and repair applications. Numerous studies have shown that interface behavior is governed by adhesion, aggregate interlock, friction, and dowel action, all of which depend strongly on the roughness and geometry of the prepared substrate [10]. In structural elements, insufficient roughening can lead to increased slippage, reduced shear transfer, and a loss of monolithic stiffness, as interface friction coefficients may vary widely, from approximately 0.4 for smooth surfaces to 1.4 for roughened ones [11]. Mechanical treatments producing surface irregularities or controlled asperities have been demonstrated to significantly enhance shear resistance by improving interlocking and reducing horizontal sliding between layers [11]. In repair scenarios, particularly in demanding hydraulic environments, proper substrate preparation is crucial to achieving reliable bonding between existing and new concrete, as roughness, cleanliness, moisture condition, and surface texture directly influence the failure mode and joint durability [12]. Beyond mechanical performance, engineered surface treatments, such as hydrophobic, pore-blocking, or coating systems, rely on the substrate’s morphological state, since pore accessibility, surface energy, and roughness govern their penetration depth and protective efficiency [13]. Together, these findings highlight that mechanical surface preparation is essential for optimizing interface performance, improving bonding, and enabling durable repairs and strengthening interventions. An additional factor in surface characterization is measurement scale. Concrete surfaces can exhibit multi-scale topography, from fine microtextures to larger asperities and undulations. Macro-scale indicators such as the International Roughness Index (IRI) are fundamental for network-level assessment, as demonstrated in Laos [14] and Taipei City [15], where the IRI supports maintenance planning, the calibration of deterioration models, and the definition of rehabilitation thresholds. However, despite its practicality and global acceptance, the IRI remains a profile-based, vehicle-response-derived indicator that captures only the overall ride quality, offering limited insight into the actual geometric morphology of the surface. This limitation becomes even more evident when roughness must be quantified at the material or interface scale, where adhesion, shear transfer, and mechanical interlocking depend on fine-scale topography. Methods such as the sand-filling test adopted in [16], which expresses roughness through the average sand height h and roughness index fi, provide only indirect scalar estimates derived from filled cavity volume and the maximum bump depth. Although useful for simple field characterization, these measures cannot capture the spatial distribution of asperities, anisotropy, or multi-scale surface features. In engineering practice, the limitations of conventional roughness indicators become even more pronounced when examining concrete-to-concrete interfaces, where roughness directly governs cohesion, frictional resistance, and adhesive bond. Current design provisions, such as EN 1992-1-1 (EC2 [17]) and Model Code 2010 (MC2010 [18]), classify interfaces into qualitative categories (“very smooth”, “smooth”, “rough”, and “very rough”) based solely on the peak-to-mean roughness parameter *Rₜ* measured with the sand-patch method. This parameter, derived by distributing a fixed sand volume over the surface and computing *Rₜ* from the resulting patch diameter, captures only a simplified macrotexture and provides no information on micro-scale asperities, anisotropy, or 3D morphological complexity. Although these classifications remain widely used in design, their single-parameter nature makes them inherently coarse and potentially unrepresentative for interfaces prepared by modern mechanical treatments. More advanced characterization is enabled by 3D digital microscopy and the family of surface descriptors defined in EN ISO 25178 [19,20]. High-resolution measurements are particularly important because many relevant surface features of concrete occur at the micro-scale. Previous studies have shown that transport processes, such as water penetration, are governed by fine matrix irregularities and micro-cracks, often with characteristic widths below 200 μm, which significantly influence long-term durability [21]. Advanced metrological research also indicates that detailed surface acquisition improves the identification of high-frequency measurement errors and enables more reliable filtering and noise reduction, especially when machine learning techniques are applied to high-resolution datasets [22]. The resolution at which measurements are performed affects the observed surface features: high-resolution measurements capture fine details that may be lost with coarser sampling, while larger intervals can smooth out surface variations and potentially underestimate surface complexity [23]. Understanding the interplay between measurement resolution and surface treatment is therefore critical for establishing standardized and reproducible assessment protocols [24]. This scale dependence is well documented across materials and surface metrology research. In pavement engineering, for example, the PIARC classification distinguishes microtexture (asperities < 0.5 mm) from macrotexture (0.5–20 mm), with each scale governing different aspects of tire–surface interaction [25]. Microtexture controls friction at the molecular contact level, whereas macrotexture affects noise, splash/spray, and rolling resistance. When measurement spacing becomes too coarse, the micro-scale asperities essential for friction and adhesion are entirely lost. A similar issue arises in concrete surface characterization. Handheld 3D scanners with a nominal precision of 0.2 mm [26] provide reliable macrotexture capture but are inherently unable to resolve the finer microtexture that governs adhesion, pore accessibility, and interface cohesion. Even when frequency-based roughness indices (e.g., Fourier Transform Roughness) are calculated, the 5 mm spacing between extracted contour lines restricts analysis to long-wavelength components, excluding critical high-frequency local irregularities. Higher-resolution laser scanning systems, sampling at 0.25 mm in x–y and 0.041 mm in z, offer a significantly improved fidelity of macrotexture representation [27], but they introduce their own uncertainties, including boundary region data loss, the need for extensive point cloud filtering, and reconstruction-induced artifacts that propagate into roughness parameters. Photogrammetry, similarly, achieves sub-millimeter accuracy only at the macrotexture scale. Even when calibrated high-resolution DSLR cameras and control points are used [28], the resulting dense point clouds inherently smooth out fine microtexture due to optical limitations and reconstruction algorithms. Semivariogram analysis confirms this: spatial autocorrelation decays rapidly with increasing lag distance, demonstrating that photogrammetric models cannot capture small-scale roughness despite high pixel resolution. Thus, semivariograms do not merely quantify spatial variability; they reveal the effective measurement resolution of each method.

Despite the recognized importance of both surface preparation and measurement scale, most studies focus on a single aspect, either the type of treatment or the measurement resolution. Limited research addresses the combined effects of different mechanical treatments and scale on the quantitative evaluation of concrete surfaces. A comprehensive analysis of both factors is essential to provide a complete understanding of surface morphology and to guide practical approaches for accurate surface characterization.

This study aims to fill this gap by systematically analyzing three surface types—patched after concreting, ground, and shot-blasted—using 3D laser scanning measurements at five scales ranging from 0.1 mm to 1.0 mm. The ground and shot-blasted surfaces represent mechanically prepared conditions, with shot blasting serving as an aggressive treatment, while the patched surface serves as a reference, reflecting the typical finishing practice in construction when no additional mechanical preparation is applied after concreting. This study evaluates how surface preparation and measurement resolution influence a comprehensive set of morphological parameters, including amplitude, statistical, volume, hybrid, and bearing descriptors, and identifies which parameters are robust to scale variation. This approach provides practical guidance for accurate and reproducible concrete surface characterization in both engineering and research contexts.

## 2. Materials and Methods

### 2.1. Three-Dimensional Laser Scanning

The surface morphology of the concrete elements was analyzed using a non-contact 3D laser scanner based on laser triangulation [29,30,31]. In this method, the deformation of a laser line projected onto the surface is measured by the scanning device. A camera, driven by a stepper motor, determines the distance between points on the scanned surface, allowing profiles to be captured with a spacing of 10 μm and an accuracy of 15 μm. The scanner was designed to minimize the number of components, facilitating its use in both laboratory and field conditions. For each measured point, three spatial coordinates are recorded, and the resulting data are saved in “.asc” format.

### 2.2. Materials

The specimens were made from a C30/37 class concrete (Gorażdże, Poland) mixture with S3 consistency, consisting of Portland cement CEM II A-LL 42.5 R, crushed basalt aggregate up to 8 mm, quartz sand up to 2 mm, fly ash, polycarboxylate-based plasticizer, and potable water, with a water-to-cement ratio of 0.48.

### 2.3. Mechanical Treatment of Samples

Concrete samples were prepared to evaluate the influence of surface treatment and measurement scale on surface morphology. Three distinct surface conditions were considered. The first, a patched surface, served as a reference and was leveled and finished manually using standard steel trowels immediately after concreting. Troweling was performed continuously until the appearance of cement laitance on the surface, at which point the process was stopped. The second, a ground surface, was obtained through mechanical grinding using a handheld angle grinder equipped with a medium-grit (80-mesh) diamond disk. Grinding was applied at a speed of ~3500 rpm, using multiple passes with light to moderate uniform pressure until a visually smooth and level surface with reduced irregularities was achieved. Each sample was ground for approximately 1–2 min, ensuring the consistent removal of surface peaks without excessive material loss. The third, a shot-blasted surface, represented an aggressive mechanical treatment and was prepared using a laboratory-scale shot-blasting machine with steel grit 0.6–0.8 mm in diameter. The grit was propelled at an air pressure of ~5 bar and a feed rate of ~1 kg/min. The process was continued for 2–3 min per sample until a pronounced textured topography and increased surface roughness were achieved. All surfaces were prepared under controlled laboratory conditions to ensure consistency across samples. Each surface type was then vacuumed before 3D laser scanning measurements.

### 2.4. Data Acquisition and Analysis

**Figure 1:**
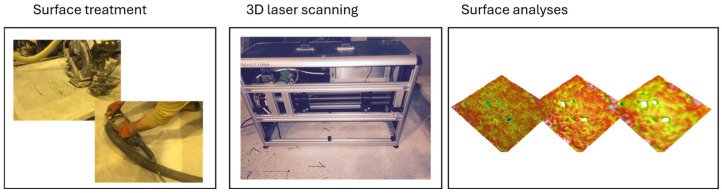
Schematical methodology of tests performed.

In the first stage of this study, concrete specimens were cast and cured under laboratory conditions. After 28 days of curing, the surfaces were subjected to mechanical treatments as described in Section 2.3, including patched (reference), ground (moderate), and shot-blasted (aggressive) preparations. Following surface treatment, 3D laser scanning was performed to obtain detailed point clouds of the surface topography. The resulting isometric images were analyzed to extract a comprehensive set of surface morphology parameters in accordance with ISO 25178 [19,32]. To evaluate the effect of measurement resolution, the same surfaces were subsequently analyzed at progressively coarser scales, decreasing the measurement resolution from 0.1 mm to 0.2 mm, 0.25 mm, 0.5 mm, and finally 1.0 mm. For each scale, the full set of morphological parameters was recalculated, allowing for a systematic assessment of how measurement scale and surface preparation influence the quantitative descriptors of concrete surface morphology. The whole process is illustrated in Figure 1.

### 2.5. Description of Selected Analyzed Morphological Parameters

The analysis of concrete surface morphology was conducted using a comprehensive set of 3D surface parameters in accordance with ISO 25178. The parameters included amplitude descriptors (*Sq*, *Sa*, *Sp*, *Sv*, *Sz*), statistical descriptors (*Ssk*, *Sku*), hybrid parameters (*Sdq*, *Sdr*), spatial distribution parameters (*Smc*, *Sxp*), volume parameters (*Vm*, *Vv*, *Vmp*, *Vmc*, *Vvc*, *Vvv*), peak and valley characteristics (*Sk*, *Spk*, *Svk*), and material ratio parameters (*Smr1*, *Smr2*). The parameters were calculated as follows.

#### 2.5.1. Height Parameters

**Figure 2:**
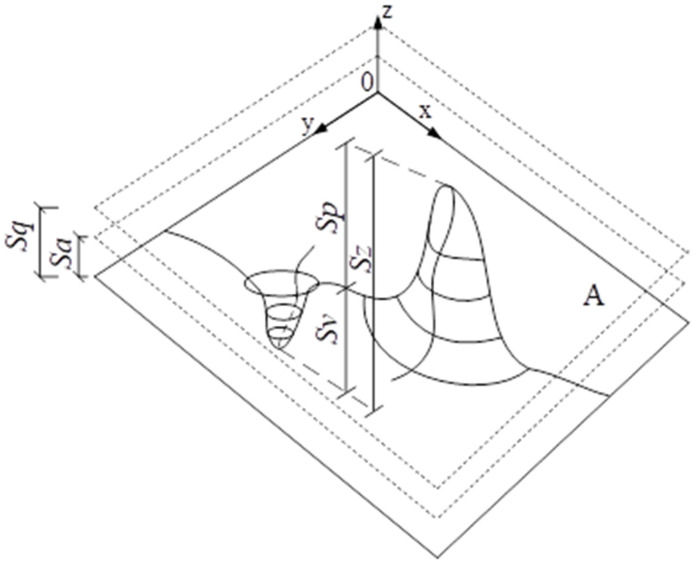
Graphical interpretation of selected height parameters.

Height parameters quantify the vertical features of the concrete surface and provide essential insights into its topography. The arithmetical mean height (*Sa*, Equation (2).) and root mean square height (Sq, Equation (1)) are particularly effective for describing the peaks, valleys, and spacing of characteristic surface elements, capturing both the average roughness and overall height variation. Skewness (*Ssk*, Equation (3)) reflects the asymmetry of the height distribution: *Ssk* > 0 indicates a surface dominated by peaks, while *Ssk* < 0 indicates a prevalence of valleys. An increase in *Ssk* corresponds to the deterioration of the surface condition, with more pronounced peaks and steep slopes, whereas a negative *Ssk* signifies a surface mainly composed of a plateau with deep and narrow valleys. Kurtosis (*Sku*, Equation (4)) provides information on the likelihood and distribution of surface defects: *Sku* < 3 indicates a low likelihood and regular distribution of defects, whereas *Sku* > 3 points to a high probability and irregular defect distribution. The maximum height parameters (*Sp*, *Sv*, *Sz*) are determined from the absolute highest and lowest points on the surface, describing the extreme peaks and valleys. Schematically, they are illustrated below in Figure 2.

(1) $$Sq=\sqrt{\frac{1}{A}\iint_{A}z^{2}\left(x,y\right)dxdy}$$

(2) $$Sa=\frac{1}{A}\int_{A}|z\left(x,y\right)|dxdy$$

(3) $$Ssk=\frac{1}{S_{q}^{3}}[\frac{1}{A}\iint_{A}z^{3}\left(x,y\right)dxdy]$$

(4) $$Sku=\frac{1}{S_{q}^{4}}[\frac{1}{A}\iint_{A}z^{4}\left(x,y\right)dxdy]$$

#### 2.5.2. Hybrid Parameters

Hybrid parameters are related to the slope of the actual surface and the increase in its area relative to the reference plane A. This group includes the root mean square gradient (*Sdq*), which quantifies the average surface slope and is dimensionless, and the developed interfacial area ratio (*Sdr*), which describes the relative increase in surface area due to texture compared to a flat reference plane, also expressed as a dimensionless value. *Sdq*, Equation (5), is calculated based on the squared gradients of the surface in both the x and y directions, while *Sdr*, Equation (6), integrates the local slope over the surface area, reflecting the complexity and functional roughness of the surface. Together, these parameters provide a measure of the surface’s three-dimensional complexity and are particularly useful for evaluating its potential contact, adhesion, and functional performance.

(5) $$Sdq=\sqrt{\frac{1}{A}\iint_{A}{\left(\frac{\partial z\left(x,y\right)}{\partial x}\right)}^{2}+{\left(\frac{\partial z\left(x,y\right)}{\partial y}\right)}^{2}dxdy}$$

(6) $$Sdr=\frac{1}{A}[\iint_{A}\left(\sqrt{{[1+\left(\frac{\partial z\left(x,y\right)}{\partial x}\right)}^{2}+{\left(\frac{\partial z\left(x,y\right)}{\partial y}\right)}^{2}]}-1\right)dxdy]$$

#### 2.5.3. Volume Parameters

**Figure 3:**
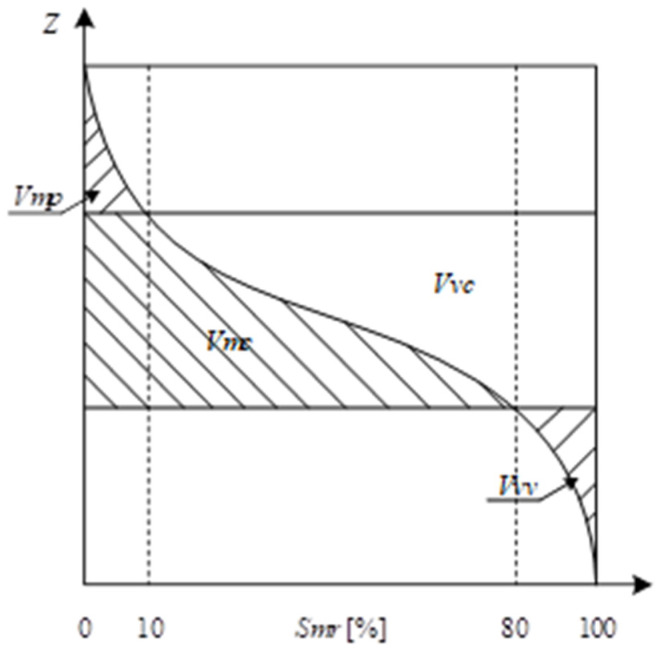
Graphical interpretation of selected volume parameters.

Volume parameters, schematically represented in Figure 3, characterize the material and void content of the surface topography, providing insight into its functional properties. This group includes *Vm*, *Vv*, *Vmp*, *Vmc*, *Vvc*, and *Vvv*, which describe the core, peak, and valley volumes of the surface. These parameters quantify the amount of material and empty space within the measured area, enabling an evaluation of the surface’s capacity to retain fluids, coatings, or adhesives. By capturing both the peaks and valleys as well as the central bulk of the surface, volume parameters offer a comprehensive understanding of surface texture and its potential impact on mechanical and functional performance.

## 3. Results

### 3.1. The Influence of the Surface Treatment Method on Morphological Parameters

**Figure 4:**
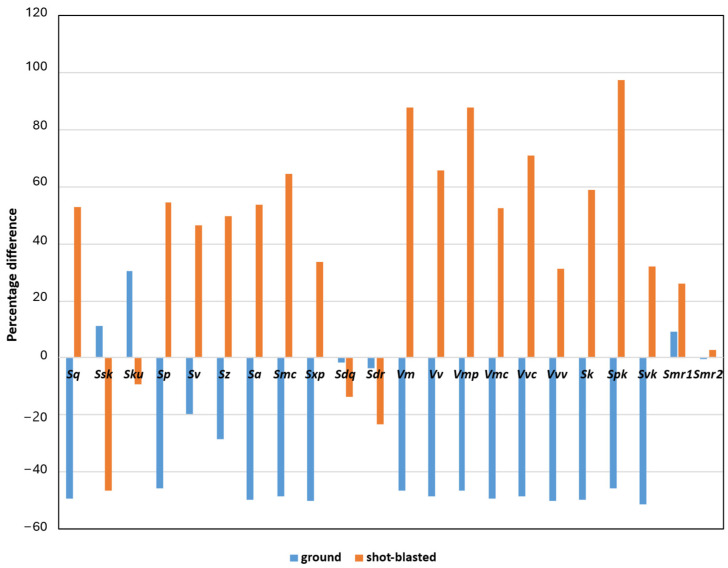
Influence of grinding and shot blasting on morphological parameters relative to patched surface after concreting.

In Table 1 and Figure 4, the differences between the values of the morphological parameters are presented with respect to the treatment method. Table 1 provides the average values calculated for each treatment type based on measurements from three samples of size 50 mm × 50 mm, while Figure 4 shows the percentage increase or decrease relative to the patched surface.

The analysis of the surface morphological parameters indicates opposite trends resulting from the two types of surface treatments compared to the patched surface after concreting. Grinding generally led to a decrease in most parameter values, particularly those describing surface height and volume characteristics (e.g., *Sq*, *Sp*, *Sz*, *Sa*, *Vm*, *Vv*, *Vmc*, *Sk*, *Svk*), with reductions reaching approximately 45–50%. This confirms that the grinding process effectively smooths and levels the surface, reducing its roughness and peak-to-valley differences. In contrast, shot blasting caused a clear increase in most of the same parameters, with some values (e.g., *Spk*, *Vm*, *Vv*, *Vvc*, *Sk*) rising by more than 50–90% relative to the untreated surface. This demonstrates that shot blasting significantly enhances the surface texture, increasing its roughness and introducing additional micro-geometry features. The only parameters showing limited or opposite variations (e.g., *Sdq*, *Sdr*, *Smr2*) suggest that certain aspects of surface slope and bearing ratio are less sensitive to these treatments.

An analysis of the statistical dispersion of the measured morphological parameters shows that the patched surface exhibits the highest variability, with standard deviations and coefficients of variation frequently exceeding 20% and in some cases surpassing 50%. This is a direct consequence of manual troweling, where maintaining uniform pressure and consistent finishing conditions across the entire surface is difficult, leading to heterogeneous compaction and localized irregularities. In contrast, both the ground and shot-blasted surfaces display substantially lower variability, typically within single-digit or low-teen CV values, reflecting the repeatable and controlled nature of mechanical treatments. The constant grinding speed and uniform abrasive action, as well as the consistent impact energy in shot blasting, ensure much more homogeneous surface morphology. These findings clearly indicate that manual finishing generates significantly greater morphological inconsistency compared to mechanically prepared surfaces.

### 3.2. The Scale Effect on Morphological Parameters

**Figure 5:**
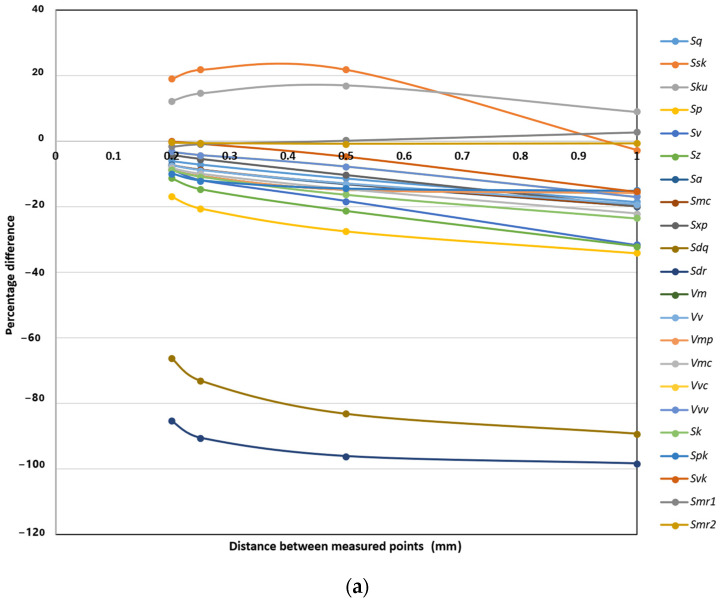

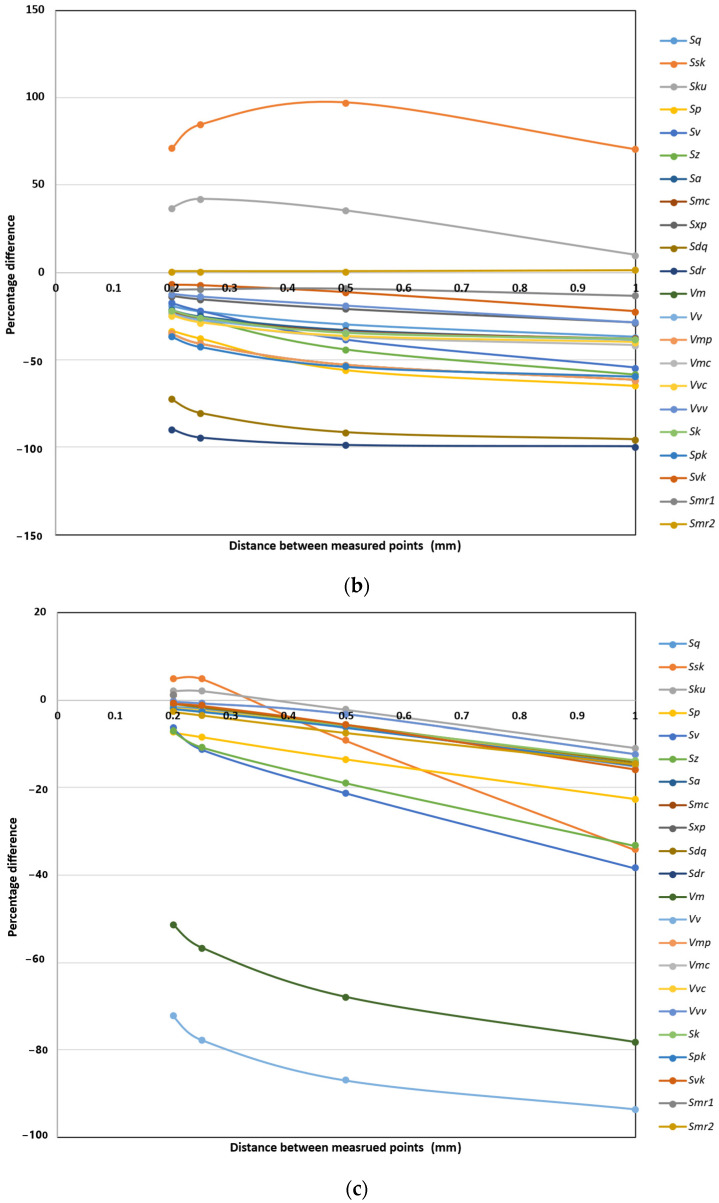
Variation in surface morphological parameters as a function of measurement scale for surfaces: (**a**) patched, (**b**) ground, and (**c**) shot-blasted.

The scale effect was assessed using five different measurement resolutions. Surface data were acquired by recording points at intervals of 0.1 mm, 0.2 mm, 0.25 mm, 0.5 mm, and 1.0 mm. For each sampling interval, all morphological parameters were calculated and subsequently analyzed to evaluate the influence of measurement scale. Figure 5a–c present the percentage decrease in the parameter values relative to the highest resolution dataset (0.1 mm) with respect to the surface treatment methods.

The analysis of the scale effect on the morphological parameters indicates that the measurement resolution has a noticeable influence on most surface characteristics for all three surface types: patched, ground, and shot-blasted. In general, as the measurement interval increases from 0.1 mm to 1.0 mm, a progressive decrease in amplitude-related parameters (*Sq*, *Sp*, *Sv*, *Sz*, *Sa*, *Sk*, *Spk*, *Svk*) can be observed. This reduction reflects the smoothing effect associated with lower-resolution sampling, where finer surface details are not fully captured. The effect is the most pronounced for the ground surface, where decreases in key roughness parameters often exceed 30–50%, confirming that ground surfaces are more sensitive to measurement scale due to their relatively fine texture. The shot-blasted surface exhibits similar trends but with slightly lower sensitivity, indicating a more uniform roughness pattern dominated by larger-scale features. The patched surface shows the mildest reduction rates, with several parameters (e.g., *Sq*, *Sa*, *Sp*, *Sv*) decreasing by less than 20% even at the coarsest scale, suggesting that its morphology is less dependent on measurement resolution. Among all parameters, *Sdq* and *Sdr* (related to surface slope and developed area) demonstrate the highest sensitivity to scale, with decreases exceeding 80–95% across all surfaces, indicating that microtexture and fine asperities are rapidly lost at lower resolutions. Conversely, some parameters such as *Ssk*, *Sku*, *Smr1*, and *Smr2* exhibit very limited variation (<5%), especially for the shot-blasted and patched surfaces, which implies that these descriptors are scale-independent within the tested range. Similarly, the volume parameters (*Vm*, *Vv*, *Vmp*, *Vmc*, *Vvc*, *Vvv*) show moderate sensitivity, typically decreasing by 10–30% for the patched surface and more significantly for the ground one.

## 4. Discussion

The findings underscore that both surface treatment methods and measurement resolution critically influence the functional interpretation of concrete surface roughness. Mechanical treatments such as grinding and shot blasting not only modify the amplitude and volumetric characteristics but also standardize the surface morphology, reducing the heterogeneity introduced by manual finishing. This has practical implications for engineering applications where consistent surface features are required, such as overlay adhesion, friction, or bonding performance. The ability to control surface morphology through the treatment method allows designers to tailor surfaces to specific functional needs, enhancing durability and performance predictability. In the literature, there are various approaches, which are presented comparatively in Table 2. 

The scale effect results highlight the importance of measurement resolution in accurately characterizing surface roughness. Coarse measurements can systematically underestimate micro-scale asperities, particularly on finely textured surfaces, potentially leading to misleading conclusions about the surface’s functional properties. Moreover, when roughness is evaluated using only 2D parameters, the results become highly sensitive to the selection of the measurement profile, which may introduce additional variability and reduce the reliability of the assessment. Parameters that remain relatively insensitive to scale, such as skewness or certain bearing ratios, may therefore serve as more robust descriptors for comparative analyses across different measurement protocols.

Therefore, the interplay between surface treatment and measurement resolution demonstrates that the functional assessment of roughness cannot rely solely on single-scale or single-parameter measurements. An accurate evaluation of adhesion, slip resistance, or mechanical interlocking requires an integrated approach that considers micro- and macro-scale features simultaneously. This reinforces the need for standardized multi-scale measurement protocols and informed surface engineering strategies to ensure that laboratory characterization translates effectively to real-world performance.

## 5. Conclusions

In this study, the morphological parameters of concrete surfaces were systematically analyzed to evaluate the influence of different surface preparation methods and measurement scales. Surfaces prepared by manual patching, grinding, and shot blasting were compared, and measurements were performed at multiple resolutions to assess their impact on the calculated surface characteristics.

1. This study demonstrated that the method of surface preparation exerts a dominant effect on the morphological parameters of concrete surfaces. Grinding significantly reduces amplitude- and volume-related parameters, producing smoother and more leveled surfaces with lower roughness and peak-to-valley values, whereas shot blasting substantially increases these parameters, generating rougher surfaces with enhanced micro-geometric features. This confirms that mechanical treatment can effectively tailor surface characteristics for specific functional or adhesive requirements.
2. Measurement resolution also significantly influences the calculated morphological parameters. As the measurement interval increases, most parameters systematically decrease due to the loss of fine surface details at coarser resolutions. Parameters related to surface slope and developed area (*Sdq*, *Sdr*) are highly scale-sensitive, showing reductions exceeding 80–90%, whereas *Ssk*, *Sku*, *Smr1*, and *Smr2* remain relatively stable (<5% variation), indicating their reliability for comparative assessments. Ground surfaces are the most sensitive to scale changes, while patched and shot-blasted surfaces show more stable responses.
3. The accurate characterization of concrete surface morphology requires consistent surface preparation combined with controlled measurement resolution. This highlights the importance of mechanically controlled surface treatments to achieve reproducible surfaces, which are critical for functional performance, including adhesion, friction, and bonding.
4. From a practical engineering perspective, the findings suggest that mechanical treatment methods should be selected and standardized according to the desired surface properties, and measurement protocols should account for scale effects to ensure reliable quality control and performance evaluation. In layered systems it is important to also take into account the type of the material that is connected to the concrete substrate, either concrete or mortar overlays of epoxy coating.
5. Future research should focus on developing multi-scale assessment protocols integrating micro- and macro-scale surface features and correlating morphological parameters with performance metrics to optimize concrete surface treatments in both laboratory and field applications, thereby enhancing both scientific understanding and practical implementation.

## Figures and Tables

**Table 1:** The average values of the morphological parameters with respect to treatment method.

Symbol of the Parameter	Patched	Ground	Shot-Blasted
*Sq* [mm]	0.242 ± 0.053	0.122 ± 0.012	0.370 ± 0.056
*Ssk* [-]	−0.790 ± 0.233	−0.878 ± 0.387	−0.423 ± 0.159
*Sku* [-]	5.373 ± 0.303	7.013 ± 3.398	4.867 ± 0.692
*Sp* [mm]	0.839 ± 0.174	0.455 ± 0.042	1.297 ± 0.131
*Sv* [mm]	1.617 ± 0.209	1.296 ± 0.690	2.367 ± 0.069
*Sz* [mm]	2.453 ± 0.293	1.750 ± 0.729	3.670 ± 0.135
*Sa* [mm]	0.182 ± 0.040	0.092 ± 0.007	0.280 ± 0.040
*Smc* [mm]	0.273 ± 0.050	0.140 ± 0.011	0.450 ± 0.060
*Sxp* [mm]	0.573 ± 0.162	0.285 ± 0.042	0.766 ± 0.175
*Sdq* [-]	1.093 ± 0.009	1.073 ± 0.606	0.943 ± 0.003
*Sdr* [%]	42.700 ± 0.497	41.067 ± 0.694	32.767 ± 0.125
*Vm* [mm^3^/mm^2^]	0.010 ± 0.003	0.005 ± 0.000	0.019 ± 0.002
*Vv* [mm^3^/mm^2^]	0.283 ± 0.052	0.145 ± 0.011	0.469 ± 0.063
*Vmp* [mm^3^/mm^2^]	0.010 ± 0.003	0.005 ± 0.000	0.019 ± 0.002
*Vmc* [mm^3^/mm^2^]	0.197 ± 0.044	0.100 ± 0.006	0.301 ± 0.038
*Vvc* [mm^3^/mm^2^]	0.246 ± 0.042	0.127 ± 0.008	0.420 ± 0.051
*Vvv* [mm^3^/mm^2^]	0.037 ± 0.011	0.018 ± 0.003	0.049 ± 0.011
*Sk* [mm]	0.524 ± 0.104	0.262 ± 0.011	0.834 ± 0.101
*Spk* [mm]	0.193 ± 0.065	0.105 ± 0.003	0.381 ± 0.047
*Svk* [mm]	0.377 ± 0.106	0.183 ± 0.035	0.497 ± 0.134
*Smr1* [%]	9.000 ± 2.054	9.810 ± 0.417	11.333 ± 0.378
*Smr2* [%]	86.900 ± 1.738	86.367 ± 0.939	89.167 ± 0.918

**Table 2:** Comparison of various scales and necessary parameters for civil engineering purposes.

Authors	Purposes	Used parameters and treatment methods	Advantages and disadvantages
Javidmehr and Empelmann [20]	Increase the bonding between the concrete substrate and UHPC	*Sz*, *Sp*, *Sv*, *Sdr*; treatment methods: brushing, sand blasting, water jetting, grooving	The usage of a few parameters to describe the surface is simple and more advanced than using just 2D parameters, but it is not well corelated with bonding strength
Farbak et al. [33]	Increase the bonding between various concretes	Coefficient of cohesion obtained by evaluation based on EN 1992-1-1 procedure and experimental tests; treatment methods: texturing with different curing conditions	The study proved that the obtained experimental values of the coefficients of cohesion are much higher (even 10 times) than assessed according to standards
AlHallaq et al. [34]	Investigating the bonding strength between the concrete substrate and the UHPC repair overlay	*Ra*, *Rz*; treatment methods: as-cast, wire brush, scarifying, scabbling	A very simple method of testing; however the correlations between parameters are not sufficient and are fully dependent on the selection of the profile tested
Zhang et al. [35]	Investigation of the bonding strength between the concrete substrate and UHPC	*Ra*, *Rq*; treatment method: texturing	Profile-based parameters are simple to evaluate and present some trends, but they are not well correlated with pull-off strength
Czarnecki et al. [36]	Investigating the bonding in repair concrete elements	*Spk*, *Sk*, *S10z*, *Sda*, *S5v*, *Sha*, *Spd*, *Shv*, *S5p*, *Sdv*, *Sz*, *Vm*, *Sq*, *Sv*, *Sa*, *Vmp*, *Vv*, *Smc*, *Vmc*, *Vvc*, *Sxp*, *Vvv*, *Sp*, *Svk*, *Spc*, *Smr1*, *Str*, and others obtained using acoustic testing combined with neural network algorithms; treatment methods: shot blasting, patching, and grinding	A very precise method of evaluating bonding; however it requires a lot of effort during the process (3 methods of testing and a prepared algorithm)
Wyjadłowski et al. [37]	Assessing the roughness of the diaphragm wall for the estimation of earth pressure	*Sq*, *Ssk*, *Sku*, *Sz*, *Sa*, *Sdr*, *Vmp*, *Vmc*, *Vvc*, *Vvv*	A very precise description of the roughness of the wall but requires special equipment

## Data Availability

The raw data supporting the conclusions of this article will be made available by the author on request.
